# A missense variant in specificity protein 6 (*SP6*) is associated with amelogenesis imperfecta

**DOI:** 10.1093/hmg/ddaa041

**Published:** 2020-03-13

**Authors:** Claire E L Smith, Laura L E Whitehouse, James A Poulter, Laura Wilkinson Hewitt, Fatima Nadat, Brian R Jackson, Iain W Manfield, Thomas A Edwards, Helen D Rodd, Chris F Inglehearn, Alan J Mighell

**Affiliations:** 1 Division of Molecular Medicine, Leeds Institute of Medical Research, Faculty of Medicine and Health, St James’s University Hospital, University of Leeds, Leeds LS9 7TF, UK; 2 School of Dentistry, Faculty of Medicine and Health, University of Leeds, Leeds LS2 9LU, UK; 3 Protein Production Facility, School of Molecular and Cellular Biology, Faculty of Biological Sciences, University of Leeds, Leeds LS2 9JT, UK; 4 Centre for Biomolecular Interactions Technology Facility, School of Molecular and Cellular Biology, Faculty of Biological Sciences, University of Leeds LS2 9JT, UK; 5 School of Molecular and Cellular Biology, Faculty of Biological Sciences, University of Leeds, Leeds LS2 9JT, UK; 6 Academic Unit of Oral Health Dentistry and Society, School of Clinical Dentistry, University of Sheffield, Sheffield, S10 2TA, UK

## Abstract

Amelogenesis is the process of enamel formation. For amelogenesis to proceed, the cells of the inner enamel epithelium (IEE) must first proliferate and then differentiate into the enamel-producing ameloblasts. Amelogenesis imperfecta (AI) is a heterogeneous group of genetic conditions that result in defective or absent tooth enamel. We identified a 2 bp variant c.817_818GC>AA in *SP6*, the gene encoding the SP6 transcription factor, in a Caucasian family with autosomal dominant hypoplastic AI. The resulting missense protein change, p.(Ala273Lys), is predicted to alter a DNA-binding residue in the first of three zinc fingers. SP6 has been shown to be crucial to both proliferation of the IEE and to its differentiation into ameloblasts. *SP6* has also been implicated as an AI candidate gene through its study in rodent models. We investigated the effect of the missense variant in SP6 (p.(Ala273Lys)) using surface plasmon resonance protein-DNA binding studies. We identified a potential SP6 binding motif in the *AMBN* proximal promoter sequence and showed that wild-type (WT) SP6 binds more strongly to it than the mutant protein. We hypothesize that *SP6* variants may be a very rare cause of AI due to the critical roles of SP6 in development and that the relatively mild effect of the missense variant identified in this study is sufficient to affect amelogenesis causing AI, but not so severe as to be incompatible with life. We suggest that current AI cohorts, both with autosomal recessive and dominant disease, be screened for *SP6* variants.

## Introduction

Enamel is nature’s most extreme example of biomineralization in humans. It results in a substance that is over 95% mineral by weight (1), a much greater content than for other examples of biomineralization, such as dentine (70%) or bone (65%) (2). Amelogenesis is the process of enamel formation. It begins with secretion by ameloblasts, the cells that form enamel, of a proteinaceous enamel matrix, created to the full thickness of the future enamel. This is then progressively mineralized through a series of repeated, cyclical processes. These involve the breakdown and removal of the enamel matrix proteins and the growth of calcium hydroxyapatite crystals to form the prisms that give enamel its hardness.

However, for amelogenesis to begin, reciprocal signaling, both to and from the future enamel-producing dental epithelium and the future dentine-producing mesenchyme, is required to initiate the final stages of pre-ameloblast differentiation into ameloblasts (3,4). This means that the formation of dentine and the initiation of amelogenesis are intrinsically linked. Pre-odontoblasts polarize, undergo internal reorganization and exit the cell cycle to become odontoblasts in response to signaling from the epithelium (5). Odontoblasts secrete an initial pre-dentine collagen matrix that, when it begins to mineralize, prompts the pre-ameloblasts to elongate and to change their polarity, so that their apical face is adjacent to the dental papilla (5). Concomitantly, the pre-ameloblasts become post-mitotic ameloblasts and secrete greater amounts of enamel matrix proteins including ameloblastin, which is thought to act as an adhesion molecule and anchor for ameloblast attachment to the secreted enamel matrix (6). The factors implicated to date in the control of the proliferation of the dental epithelium and the differentiation of ameloblasts are numerous and include both the RUNX2—NFIC—OSX (also known as SP7) transcription factor pathway (7), the Sp6 transcription factor (8) and many other transcription factors and signaling molecules. SP6 is also known as specificity protein 6 or epiprofin and was previously called Krüppel-like factor 14 (KLF14).

Amelogenesis imperfecta (AI) is a heterogeneous group of genetic conditions characterized by defective enamel. AI can be broadly classified based on the enamel phenotype, although mixed phenotypes do occur. Defects at the start of or during enamel matrix secretion tend to cause hypoplastic AI, where the enamel is absent or thin and variably mineralized. Defects during the maturation stage generally result in hypomineralized AI, where the enamel is of full thickness but is weak and inevitably fails prematurely. Hypomineralized AI has been further subdivided into hypomaturation and hypocalcified AI that produce brittle and soft enamel, respectively. AI may present as an isolated phenotype or may be associated with other oral or extra-oral features as part of a syndrome. The prevalence of AI has been reported to be 1 in 700 in an isolated Swedish population (9) and around 1 in 14 000 in the US population (10).

Mutations in many genes are known to cause AI, and these can be inherited in an autosomal recessive, dominant or X-linked manner (11). Mutations in the genes encoding the enamel matrix proteins (*AMELX*, *AMBN* and *ENAM*) and the enamel proteinases (*MMP20* and *KLK4*) were the first to be reported to cause AI. Variants in other genes encoding proteins that mediate or affect cell adhesion (*LAMA3*, *LAMB3*, *COL17A1*, *FAM83H* and *ITGB6)* or are thought to be involved in endocytosis, calcium transport and pH sensing (*WDR72*, *SLC24A4* and *GPR68,* respectively) have also been implicated. Mutations in the gene encoding transcription factor DLX3 and in genes encoding proteins for which their function in amelogenesis is less clear (e.g. *ODAPH*, *AMTN*, *ACP4, RELT* and *FAM20A*) are also known to cause AI. Despite this, between 51 and 72% of AI cases are reported to be genetically undiagnosed (12–14). However, mutations in many more genes have been identified as a cause of AI since some of these studies were published and massively parallel sequencing is in now routine use, so the detection rate at present is likely to be higher than in those reports.

Here we report a family with dominantly inherited, hypoplastic AI carrying a variant in the Sp6 transcription factor gene (*SP6*). SP6 has been shown to be involved in ameloblast differentiation (15) and regulation of tooth-related genes (16). It has also been shown to be expressed during the secretory stage of amelogenesis (17) and in the pre-ameloblast inner enamel epithelium (IEE) (18). We model the effect of the variant on protein function and analyze its impact on SP6 binding to target gene promoters.

## Results

### Patient phenotype

**Figure 1:**
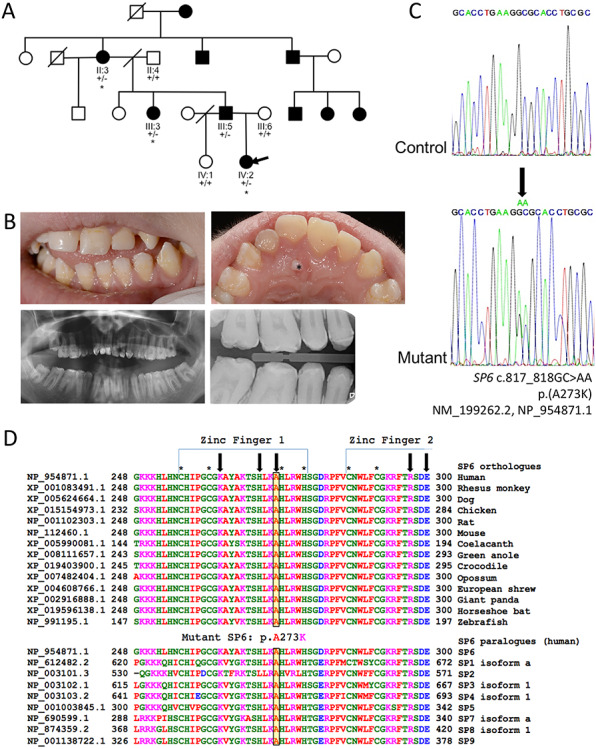
Family pedigree, dental phenotype, genotyping and conservation. (**A**) Pedigree of the British family investigated. Affected family members are shaded. WES was carried out on the individuals marked with asterisk. Segregation analysis of the *SP6* c.817_818GC>AA for all available family members is also shown. (**B**) The permanent dentition of the index case, IV:2 (arrow on pedigree) was characterized by generalized hypoplastic AI with an irregular surface involving all teeth. Note: the small soft tissue lesion involving the hard palate (marked with asterisk) is a reactive lesion unrelated to the dentition. (**C**) Sanger sequencing electropherograms to show the WT SP6 and the *SP6* c.817_818GC>AA (NM_199262) variant sequence. (**D**) Conservation analysis of the p.Ala273 residue in orthologous and paralogous proteins.

We identified a White British family segregating autosomal dominant hypoplastic AI in the absence of any clinically obvious co-segregating health problems (Figure 1A).

### Whole-exome sequencing, PCR and Sanger sequencing

To identify the cause of AI in this family, we performed whole exome sequencing (WES) on DNA from individuals II:3, III:3 and IV:2 (Figure 1A). Following alignment, processing and duplicate removal, a mean depth of 56.71, 64.12 and 87.62 reads per base was observed, respectively, for individuals II:3, III:3 and IV:1 with 98.3, 98.5 and 99.0% of bases covered by at least 5 reads, respectively, (further alignment statistics are available in Supplementary Material, Table S1). Indel and single nucleotide variants were called in variant call format (VCF) using the Haplotype Caller function of Genome Analysis Toolkit (GATK) (19).

Variants were selected based on the autosomal dominant inheritance evident in the family (confirmed by male-to-male transmission). The resulting variants were then filtered to select those present in all three affected individuals (II:3, III:3 and IV:2) and to exclude all changes other than missense, frameshift or stop mutations, exonic insertions/deletions or variants located at splice consensus sites.

In addition, variants present in gnomAD (v2.1.1) (20) were excluded if present at a frequency higher than that determined using the allele frequency app (http://cardiodb.org/allelefrequencyapp/) (21). A filter frequency cutoff of 4.51 × 10^−5^ was obtained using the following input values (data accessed May 1, 2017): monoallelic disease, 1 in 700 prevalence (the highest prevalence reported for AI (9)), an allelic heterogeneity value of 0.06 based on 132 reported autosomal dominant families, and the most frequently reported variant, *ENAM* c.1259_1260insAG, having been reported in 7 families to date (11). Genetic heterogeneity was arbitrarily set to 1 as per app instructions.

This left 12 variants, of which only 8 segregated with the disease phenotype after PCR and Sanger sequencing of DNA from all available family members and segregation analysis (Supplementary Material, Table S2). One further variant (chr17:43553034G>A) in *PLEKHM1* could not be confirmed nor checked for segregation with disease due the presence of a near identical pseudogene, *PLEKHM1P*. It is noteworthy that a rare SNP (rs768117863) is present at the homologous position for the variant in *PLEKHM1P* (chr17:62818453G>A). The sequences of the two homologous exons are 98.8% identical, suggesting that alignment quality might be compromised for these positions.

Alongside variant calling, copy number variant (CNV) analysis was also performed, using ExomeDepth software (22). This compares read depths across all captured exons of samples from affected individuals (II:3, III:3 and IV:2) against the read depths of 10 samples from unrelated individuals whose DNA had been processed within the same WES batches, using identical conditions, as the affected samples from the family. After filtering to select only CNVs that occur in all three affected family members and that were not also called in three unrelated individuals, one rare CNV (not in the Database of Genomic Variants (23)) in *SIGLEC11* remained (Supplementary Material, Table S3).

Variant filtration in this family therefore appeared to exclude the involvement of all AI genes known to date, suggesting the involvement of a variant in a gene not previously implicated in AI. Supplementary Material, Table S4 reviews the available literature on the potential for involvement in AI of each of the 10 remaining candidate genes and variants. *CALHM3*, *PCK2*, *KRT76*, *NME8*, *RAB26* and *SIGLEC11* are relatively tolerant of variation (gene missense *Z* score ≤ 0.35) making it unlikely that heterozygous variants in these genes could cause Mendelian disease. For five of these six candidates, there is no known involvement in inherited disease of any sort, but polymorphisms in *CALHM3* have been associated with susceptibility to Alzheimer disease (24) and Creutzfeld-Jakob disease (25) and are known to be involved in taste perception (26). Of the four remaining genes, human disease is already associated with variants in *PLEKHM1* (OMIM #611497 osteopetrosis) and *EPOR* (OMIM #133100 erythrocytosis), but the family presented here does not exhibit either of these phenotypes. Very little is known about *EFCC1*, but again there is no known link with tooth development or function or involvement in any form of disease phenotype, either in humans or in animal models.

Variants in the tenth candidate gene, *SP6,* have also not been associated with disease in humans to date. However, SP6 is known to have an essential role in ameloblast differentiation and to regulate the expression of many tooth-related genes. Furthermore, *SP6* is already implicated in AI in a rat model carrying an *Sp6* 2 bp insertion (27,28) and in two murine *Sp6*^−/−^ models (15,29). It has also previously been proposed as a candidate gene for AI (Supplementary Material, Table S4) (28). Therefore, of the variants that remained after segregation, the two base pair missense variant in *SP6*, c.817_818GC>AA, p.(Ala273Lys) (NM_199262.2, NP_954871.1) was prioritized for further investigation. This variant was absent in dbSNP150 and Genome Aggregation Database v.2.1.1 (gnomAD; accessed 08/02/2019). The variant is predicted to affect a residue that lies within the first of three C_2_H_2_ zinc finger domains and is highly conserved in SP6 orthologues in all species analysed and in all other SP family paralogues (Figure 1B). The p.(Ala273Lys) substitution is predicted to be deleterious or damaging by Provean, SIFT and Polyphen-2 with a combined annotation-dependent depletion (CADD) (v1.3) score of 33, suggesting that protein structure and/or function may be affected.

Based on this result, we screened all known exons and flanking intronic sequence of *SP6* (based on NM_001258248 and NM_199262) in a further 35 dominant AI samples, but no single nucleotide variants nor small indels in *SP6* were identified (Supplementary Material, Table S5).

### Protein structural analysis

Literature searching and database interrogation showed that SP6 consists of 376 amino acids and contains three ZnF domains (His254 to His278 [25aa], Phe284 to His308 [25aa] and Phe314 to His336 [23aa]) responsible for DNA binding (30). Within these three ZnF domains, three particular residues per ZnF contact the DNA. The variant identified here (p.(Ala273Lys)) alters a residue in the first ZnF domain that is predicted to form a direct contact with target DNA sequences (31). Therefore, p.(Ala273Lys) is likely to cause disease by affecting the binding of mutant SP6 to DNA.

We searched the Protein Data Bank (PDB) for high-resolution structures for SP6. Although SP6 does not currently have a structure file in PDB, there are NMR structures of the three ZnFs of the highly homologous SP1 protein (Figure 1D). These structures (PDB codes 1VA1, 1VA2 and 1VA3) show the position corresponding to p.Ala273 on the solvent-exposed face of the ZnF α-helix. Substitution with a large, polar side chain in this location is unlikely to perturb protein folding as packing of the hydrophobic core or zinc ion binding is unaffected.

Crystal structures of other Cys2His2 ZnF-DNA complexes, for example, for GLI and ZIF268 (PDB codes 2GLI and 1ZAA, respectively) allowed us to assess likely effects of the p.(Ala273Lys) variant. These show the α-helix docking into the major groove with contacts from various positions along the helix. Some fingers show contact to DNA from the amino acid immediately N-terminal to the His2 motif, including His, Val, Thr, Arg and indeed Lys (Supplementary Material, Figure S1). With the C-terminus of the α-helix (and the His2 motif) being relatively solvent-exposed and with the flexibility of the lysine side chain, it is not clear whether the increased volume of the p.(Ala273Lys) change would strongly inhibit docking of the SP6 mutant protein with DNA.

### Identification of candidate SP6 promoter motifs and binding assays

Since SP6 is a transcription factor, we tested the effect of the c.817_818GC>AA, p.(Ala273Lys) variant on target sequence specificity and DNA binding through Biacore surface plasmon resonance (SPR) binding assays. Searching JASPAR (32) showed that the binding motif for SP6 is not known, but binding motifs for SP1, 2, 3, 4 and 8 are reported (Supplementary Material, Table S6). SP proteins are known to bind CG-rich promoter elements in promoter proximal regions (33) and the motifs identified in our search included GC boxes (GGGCGG), although these were generally identified on the non-coding strand. We then searched the proximal promoter sequences of known rodent SP6 target genes *Amtn*, *Rock1*, *Car3*, *Fst*, *Osr2* and *Pcm1* (Utami *et al* (16)), for sequences similar to the DNA motifs recognized by other SP proteins. We identified a 9 bp CCCCGCCCC motif, which contains the GC box sequence in antisense, within 101 bp or fewer upstream of the transcriptional start sites of the human genes *ROCK1*, *CA3*, *FST* and *PCM1*. Alternative shorter anti-sense GC box-containing sequences and other CG rich regions were identified upstream of the transcriptional start site of *OSR2* (e.g. CCCGCCC). No motif was identified within the proximal promoter region of *AMTN*. The CCCCGCCCC motif has been reported previously to be bound by SP1 (34) and has been associated with nucleosome remodeling (35). This 9 bp motif should have a 50% chance of occurring randomly once every 181 Kbp.

Another report presents evidence that SP6 may regulate AMBN expression in mice, although direct binding of SP6 to the *Ambn* promoter region was not assessed (15). We searched the proximal promoter region of *AMBN* and identified an 8 bp motif CCCGCCCC, similar to the 9 bp motif identified in the other genes, at 97 bp upstream from the transcriptional start site. Interestingly, this motif is within 4 bp of a second distal GC-rich motif CCCCCCCGCCAC.

**Table 1:** Affinity and kinetic values describing SP6-DNA binding to the *AMBN* oligonucleotide by Biacore SPR

	*K_D_* (nM)	*k_a_* (M^−1^ s^−1^)	*k_d_* (s^−1^)
WT SP6	226 (±12)	2.18 × 10^4^ (±0.005 × 10^4^)	4.91 × 10^−3^ (±0.18 × 10^−3^)
Mutant SP6	295 (±77)	2.07 × 10^4^ (±0.053 × 10^4^)	5.68 × 10^−3^ (±0.18 × 10^−3^)

Values were determined by data fitting for all concentrations tested over 3-min injections and 5 min of dissociation (*n* = 3)

**Figure 2:**
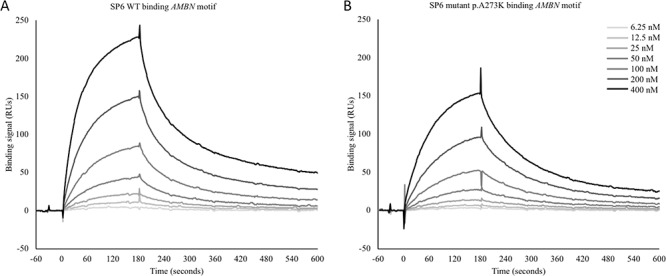
Comparison of DNA-binding activity of WT and mutant SP6 proteins using Biacore SPR. Biotinylated oligonucleotides were captured on a streptavidin–derivatized sensor chip surface. WT and mutant SP6 proteins were washed over these surfaces across a range of concentrations. Each 3-min injection was followed by buffer washes to follow dissociation rates of the SP6-DNA complex. Sensorgram results are shown for the *AMBN* oligonucleotide (sequence in Supplementary Material, Table S8) (**A**) WT SP6; (**B**) mutant SP6. DNA-binding by the mutant SP6 protein demonstrates that this variant does not abolish binding, consistent with the mutation affecting only one of the three zinc finger motifs. However, the mutant does show reduced binding compared to the WT, with the dissociation rate for the mutant SP6 protein being faster than for the WT. This is clear on the *AMBN* promoter sequence surface with the signal dropping to lower values than for the WT protein.

In addition, we searched the proximal promoter regions of other known and candidate AI genes, as well as *SP6* itself, for potential SP6 binding sites. GC-rich motifs were identified in the proximal promoter regions of *FAM83H* and *SP6*. No likely motifs were identified in the promoters of known and candidate AI genes *ACP4*, *AMELX*, *COL17A1*, *DLX3*, *ENAM*, *FAM20A*, *GPR68*, *ITGB6*, *KLK4*, *LAMA3*, *LAMB3*, *MMP20*, *ODAM*, *ODAPH*, *RELT*, *SLC10A7*, *SLC24A4*, *WDR72* and *TUFT1*. All motifs identified are detailed in Supplementary Material, Table S7.

Four biotinylated complementary probes were designed for Biacore SPR studies of protein-DNA interactions (Supplementary Material, Table S8). The first three contain a putative SP6 binding motif from known SP6 bound target gene promoters; *ROCK1* (chr18:18691847–18691886 negative stand) (16,36), *AMBN* (chr4:71457862–71457901 positive strand) (15) and *CA3* (chr8: 86350989–86351028 positive strand) (16). The fourth contains a region, GCCTCCGGGAGGC, known to bind SP3 within the *NKX2-1* promoter (chr14:36989530–36989569) (37) and was included to test SP6 specificity.

To study the effect of the p.(Ala273Lys) variant *in vitro*, we cloned and expressed WT and mutant SP6 proteins. SPR was carried out using the four probes and a control underivatized surface as a reference flow cell. SP6 WT and SP6 p.(Ala273Lys) mutant proteins were injected over derivatized surfaces followed by buffer to observe the binding and dissociation rates of the DNA-protein complexes.

Neither WT nor mutant SP6 protein bound the *NKX2-1* promoter sequence (data not shown), supporting specificity for an exclusively-GC SP6 binding motif. Both proteins bound the *ROCK1* and *CA3* promoter sequences but gave low signals: 16 response units (RU) and 29 RU at maximum, respectively, for the 400 nM WT protein condition (Supplementary Material, Figure S2). In contrast, strong binding was evident for WT and mutant SP6 with the *AMBN* promoter sequence (228 RU and 154 RU, respectively, at maximum for the 400 nM protein concentration), as shown by the increasing signal with increasing concentration (Figure 2, Additional File 1). This shows that this oligonucleotide contains a relevant binding sequence for WT SP6. DNA-binding by the mutant protein demonstrates that this allele does not abolish this binding, consistent with the mutation affecting only one of the three zinc finger motifs. However, the mutant does show reduced maximal binding compared to the WT, a reduction of around 32%. The difference in binding is largely due to a significantly faster dissociation rate for the mutant protein-DNA complex than the WT (Table 1). This is clear for the *AMBN* promoter sequence surface, since the signal dropped at a faster rate and to lower values than for the WT protein.

## Discussion

We identified a family with autosomal dominant hypoplastic AI in whom WES revealed a 2 bp variant, c.817_818GC>AA in *SP6,* resulting in the missense change p.(Ala273Lys). PCR analysis confirmed that the *SP6* variant segregates with the disease phenotype along with eight other variants. A screen of 35 additional dominant AI families revealed no further variants in *SP6*.

SP6 belongs to the SP transcription factor family and consists of a proline rich N-terminal domain and a C terminal domain containing three C_2_H_2_ zinc finger domains (31). The zinc finger domains are believed to contact DNA via specific residues (38). For the first of the three zinc fingers in SP6, these residues are predicted by homology to be Lys267, His270 and Ala273 (31). The variant identified in the family reported here changes the small hydrophobic Ala273 residue to a large, hydrophilic residue, potentially altering the structure of the first zinc finger domain and also its function.

Mutations in other SP family proteins have not been reported except for *SP7*. A frameshift mutation in *SP7* was reported in a single case with autosomal recessive osteogenesis imperfecta type XII (MIM #613849) (39). Similarly, three siblings with the same disease were reported with a homozygous c.946C>T variant (40). This results in missense change p.(Arg316Cys) within the first zinc finger of SP7, similar to the position of the missense variant in SP6 identified in this study (for reference, the equivalent SP6 residue is p.Arg276). It is notable that one of the heterozygous carrier parents also met the criteria for adult osteoporosis, suggesting that missense variants affecting this part of the protein may be sufficient to cause mild disease even when one WT copy is present.

Rodent models of SP6 function include two murine SP6 null (*Sp6*^−/−^) lines (15,29) and a rat (*Ami*/*Ami*) with a frameshift mutation in the third zinc finger domain (NM_001108833.1: c.965_966insGT, NP_001102303: p.F323Sfs^*^12). The *Sp6*^−/−^ mice show either total absence of enamel or secretion of a thin irregular enamel layer (29). The *Ami/Ami* rats have hypoplastic AI (28), a similar phenotype to that of the family presented here. The *Sp6*^−/−^ mice also display a range of other phenotypes including delayed tooth eruption, supernumerary teeth, fused teeth, defective cusp formation, malformed roots and enlarged dentine tubules as well as retarded growth, failure to develop fur and abnormalities in limb development and lung alveolarization (15,29). The phenotype of the *Ami*/*Ami* rat model appears to be more restricted to enamel formation, although at birth, whiskers are curly and are weak throughout adulthood (28). Heterozygous *Sp6*^+/−^ or *Wt*/*Ami* animal models do not display an AI phenotype, but *Sp6*^+/−^ mice have been reported to have a wider enamel layer with an irregular and less compact structure than WT (15). *SP6* has previously been highlighted as a candidate gene for human AI, although autosomal recessive inheritance was predicted based on the animal models (28). Based on the information from animal models, the mutation itself appears critical to the range of phenotypes seen and their severity. The missense variant reported here may not be accurately modelled by the *Sp6*^−/−^ mouse or the *Ami*/*Ami* rat. It is also possible that a gain of function may occur if DNA binding specificity or ability is altered by the p.Ala273Lys substitution, as it does appear to be from SPR studies.

Studies of *Sp6*^−/−^ mice suggest that the function of SP6 in amelogenesis is dependent upon the developmental stage. Firstly, SP6 promotes proliferation of the IEE, and secondly, it stimulates the differentiation of these cells to form ameloblasts (15). One murine *Sp6* transcript (NM_031183) has been detected in the posterior neuropore, the apical epidermal ridge of limb buds and in teeth and hair follicles of murine embryos but not in adult tissues (41), suggesting that the function of this *SP6* transcript may be entirely developmental. The expression of the other murine transcript, NM_001363230, which codes for an identical protein, has been described as ubiquitous (31) although an antisense transcript has also been detected, which may serve to regulate expression levels, so that embryonic expression of the protein is significantly higher than in the adult. Unfortunately, no relative quantification of the three transcripts’ expression levels in embryonic dental tissues has been demonstrated to date, meaning that it is unclear whether SP6 is expressed in adult tissues.

Our attempts to examine the effect of the mutation on the binding of SP6 to its target sequences have been hampered by lack of knowledge of the sequences SP6 binds within its target proximal promoter regions. Literature searching showed that SP6 probably binds GC rich motifs such as GC boxes, and SPR studies supported this. Of the motifs tested, the *AMBN* proximal promoter sequence was most strongly bound. The binding for the *ROCK1* and *CA3* promoter sequences was much lower in comparison, perhaps suggesting that the proximity of other GC rich sequences or the surrounding sequence context affects binding efficiency. In addition, the lack of GC-rich sequence in the *AMTN* proximal promoter region suggests that there might be additional sequence motifs to which SP6 binds or that it might influence expression through other intermediates.

The *AMBN* promoter sequence was most strongly bound by WT SP6. In comparison, mutant SP6 protein also bound the oligonucleotide sequence but less strongly and it dissociated faster than the WT protein. This indicates that while binding was affected by the variant, its negative effect may be relatively mild. Given the likely pleiotropic effect of a more damaging variant on the action of a transcription factor involved in development of multiple tissues and organs such as SP6, this may explain why further variants in *SP6* causing AI or other human phenotypes have not been reported to date. A more damaging variant may not be compatible with life. Interrogation of gnomAD identified only 9 high quality loss of function variants in 10 individuals, with the highest reported allele frequency being 1.549 × 10^−5^. Only three missense variants, (p.Thr77Asn, p.His159Tyr and p.Glu337Gln) were reported to have been identified as homozygous, each in only one individual. None of these variants are within the three zinc fingers domains, again highlighting the importance of these domains to SP6 function.

Results from the SPR study suggest a mechanism by which the c.817_818GC>AA *SP6* variant may cause AI. Reduced binding of mutant SP6 to the *AMBN* proximal promoter sequence *in vivo* could reduce the transcription of *AMBN* during amelogenesis, resulting in less AMBN protein present in the enamel matrix. Perturbation of AMBN levels has been shown to affect the levels of other proteins important in amelogenesis, for example, MSX and AMELX (42) and to affect ameloblast adhesion to the extracellular matrix (43). These alterations are known to result in hypoplastic AI. Other SP6 transcriptional targets, in addition to *AMBN*, may also be affected. Investigation of the effects, of either the rat or murine *Sp6* variants, on AMBN expression in developing tooth buds from these models could be useful in determining whether the location, timing or level of AMBN expression is altered.

In conclusion, a missense variant in *SP6* has been found to segregate with AI in a family with autosomal dominant inheritance. Given the prior evidence for the role of SP6 in amelogenesis, the phenotype observed in three rodent models and our data on the impact of this variant on binding to a likely target DNA sequence of a known AI disease gene encoding a protein critical for correct enamel formation, we suggest that this missense variant is almost certainly the causative variant in the family described here. Nevertheless, this finding would benefit from replication in other cohorts, making *SP6* a strong candidate gene for further screening in AI. However, we further hypothesize that the relatively mild effect of the missense variant shown in this study is sufficient to cause AI but not so severe as to be incompatible with life, which may explain why *SP6* variants are rare as a cause of AI.

## Materials and Methods

### Patients

Affected individuals and family members were recruited following informed consent in accordance with the principles outlined by the declaration of Helsinki, with local ethical approval. Genomic DNA samples were obtained using Oragene^®^ DNA sample collection kits (DNA Genotek, ONT, Canada) according to the manufacturer’s instructions.

### WES and analysis

Three micrograms of genomic DNA were processed according to the Agilent SureSelect XT Library Prep protocol (Agilent Technologies, CA, USA). Sure Select Human All Exon V5 or V6 (Agilent Technologies) was used as the capture reagent. Sequencing was performed using a 150 bp paired-end protocol on an Illumina HiSeq 3000 sequencer (4981 and 4982) (Illumina, CA, USA). The resulting fastq files were aligned to the human reference genome (GRCh37) using BWA (44). The alignment was processed according to GATK best practice. Exome depth was used for CNV analysis according to the developers’ guidelines (22).

All genomic coordinates are based on the GRCh37 human reference genome. The reference gene sequence upon which *SP6* mutation nomenclature is based is RefSeq transcript NM_199262.

The variant identified in this study has been submitted to the Leiden Open Variant Database at http://dna2.leeds.ac.uk/LOVD/ variant ID: 0000000305.

### Protein synthesis

The SP6 wild type and mutant (p.A273K) coding sequences were synthesized (GeneWiz, South Plainfield, NJ, USA) in pUC57 with codon optimization for *E. coli* expression and then PCR-amplified using Q5 DNA polymerase (NEB, Evry, France; 5′ primer: AAGTTCTGTTTCAGGGTACCATGCTGACCGCCGTTTGTGGC, 3′ primer: CTGGTCTAGAAAGCTTTTAATTGCTCGGGGCAACGC). Both were cloned into pOPINJ using the NEBulider HiFi assembly to produce constructs pOPINJ_SP6 and pOPINJ_SP6mut, each containing an N-terminal His-GST tag.

pOPINJ_SP6 was transformed into BL21 (DE3) pLysS (Agilent Technologies, Santa Clara, CA, USA), grown in terrific broth (TB with 100 μg/ml ampicillin and 34 μg/ml chloramphenicol) to an OD_600_ of 0.6, induced with 0.4 mm IPTG and 10 μM ZnCl_2_ and incubated for 18 h at 27°C. Bacteria were harvested through centrifugation at 4000*g* for 40 min, the pellet resuspended into PBS + 1% Triton X-100 and the bacteria lysed using sonication (10 s on, 30 s off, for 10 bursts at amplitude 60%). The soluble lysate was purified on an AKTA Pure (GE Healthcare, Little Chalfont, UK) using nickel affinity chromatography with a HisTrap HP 5 ml column into His elution buffer (20 mm Tris, 150 mm NaCl, 5% glycerol and 400 mm imidazole, pH 7.6) using a gradient elution. His-GST-SP6 was further purified through ion exchange on a HiTrapQ HP 1 ml column and eluted with a gradient elution in 10 mm HEPES, 1 M NaCl, pH 7.5. Resulting elutions were concentrated in a Pierce protein concentrator (10 K MWCO), and the concentrated protein was aliquoted, frozen in liquid nitrogen and stored at −80°C.

pOPINJ_SP6mut was transformed into ArcticExpress (DE3) cells (Agilent Technologies) and grown in TB (with 100 μg/ml ampicillin and 50 μg/ml gentamicin) for 3 h at 30°C. Cultures were then equilibrated for 10 min at 12°C prior to induction with 0.4 mm IPTG and 10 μM ZnCl_2_ and incubated for 24 h at 12°C. Bacteria were harvested through centrifugation at 4000*g* for 40 min, and then the pellet was resuspended into lysis/wash buffer (50 mm Tris, 300 mm NaCl, 20 mm imidazole, 5% glycerol, pH 7.6). The bacteria were lysed using sonication as before. The soluble lysate was purified using Ni^2+^ NTA agarose beads into His elution buffers with varying imidazole concentration using a step elution. Soluble His-GST-SP6mut protein was identified at 80 and 160 mm imidazole and pooled. His-GST-SP6mut was further purified through ion exchange on a HiTrapQ HP 1 ml column and eluted with a gradient elution in 10 mm HEPES, 1 M NaCl, pH 7.5. Resulting elutions were concentrated in a Pierce protein concentrator (10 K MWCO), and the concentrated protein was aliquoted, frozen in liquid nitrogen and stored at −80°C.

### DNA probe preparation

Four probes were designed for protein-DNA interaction studies using SPR. Single forward strand DNA molecules were synthesized and biotinylated (Sigma–Aldrich) on the 5′-end (Supplementary Table S8). Reverse complementary strands were synthesized without biotinylation.

DNA oligos were dissolved at 100 μM in TE buffer. Single-strand biotin-labelled and non-biotin-labelled DNA oligos were diluted in TM buffer [10 mm Tris (pH 7.5) and 10 mm MgCl_2_] to a final concentration of 10 μM, annealed at 95°C for 1 min and cooled slowly to room temperature. Annealed DNA probes were kept at −20°C before use.

### Surface plasmon resonance

SPR experiments were performed on a Biacore 3000 instrument (GE Healthcare). Biotinylated DNA probes at 10 nM were immobilized on streptavidin (SA) sensor chips (GE Healthcare) at a flow rate of 5 μL/min, to give ∼500 response units (RU) of immobilized DNA. The reference flow cell was underivatized. All ligand immobilization was done in HEPES-buffered saline consisting of 10 mm HEPES (pH 7.5), 200 mm NaCl and 0.01% (v/v) surfactant P-20. Analyte measurements were carried out at 25°C and a flow rate of 40 μL/min, using the same buffer. For SP6–DNA-binding assays, 120 μL of IEX-purified SP6 (WT or p.A273K) was injected across flow-cell surfaces in a two-fold ascending concentration series, from 6.25 to 400 nM for 3 min. Subsequently, buffer was washed over the surfaces to observe the dissociation rates of the DNA-protein complexes. The chip surface was regenerated between protein injections with a 40 μL 0.05% (w/v) SDS injection. Binding data were processed using double-referencing by subtraction of signals from a reference flow cell and by subtraction of a buffer injection over each derivatized flow cell. Binding data were analyzed using BIAevaluation 3.1 software (GE Healthcare).

## Supplementary Material

SP6_Supplementary_data4_ddaa041
