# Supplementary material for: A missense variant in specificity protein 6 (SP6) is associated with amelogenesis imperfecta
Source: Hum Mol Genet. 2020 Mar 13;29(9):1417–25. doi: 10.1093/hmg/ddaa041 (PMC7268548; doi:10.1093/hmg/ddaa041)
Supplement: SP6_Supplementary_data4_ddaa041 [file sp6_supplementary_data4_ddaa041.docx]

**Supplementary data**

| **Sample** | **Capture reagent** | **Depth of coverage** | **% bases covered by >4 reads** | **% bases covered by >9 reads** | **% bases covered by >14 reads** | **% bases covered by >19 reads** | **% bases covered by >24 reads** | **% bases covered by >29 reads** |
| --- | --- | --- | --- | --- | --- | --- | --- | --- |
| II:3 | SSV6 | 56.71 | 98.3 | 94.8 | 89.8 | 83.7 | 77.2 | 70.4 |
| III:3 | SSV6 | 64.12 | 98.5 | 95.5 | 91.4 | 86.3 | 80.7 | 74.9 |
| IV:2 | SSV5 | 87.62 | 99.0 | 98.4 | 97.5 | 96.2 | 94.2 | 91.7 |

**Table S1** Alignment statistics for whole exome sequencing.

Alignment statistics were generated using the regions targetted by the capture reagent: SSV6 - SureSelectXT Human All Exon V6; SSV5 - SureSelectXT Human All Exon V5.

| **Genomic variant (GRCh37)** | **dbSNP reference^a^** | **cDNA variant and predicted amino acid change** | **Gene** | **CADD v1.3^b^** | **SIFT^c^** | **Polyphen2^d^ (HumVar)** | **RefSeq**  **transcript** |
| --- | --- | --- | --- | --- | --- | --- | --- |
| 17:45924978_ 45924979GC>AA | N/A | c.817_818GC>AA  p.(A273K) | *SP6* | 33 | 0  Damaging | 1  Probably Damaging | NM_199262.2, NP_954871.1 |
| 17:43553034G>A | N/A | c.355C>T  p.(R119W) | *PLEKHM1* | 29.7 | 0  Damaging | 0.909  Probably Damaging | NM_014798.3  NP_055613.1 |
| 10:105233334T>G | N/A | c.671A>C  p.(Q224P) | *CALHM3* | 27.5 | 0.015 Damaging | 0.862  Possibly Damaging | NM_001129742.1  NP_001123214.1 |
| 3:128720890T>G | N/A | c.419T>G  p.(L140R) | *EFCC1* | 25.2 | 0.001 Damaging | 0.973  Probably Damaging | NM_024768.2  NP_079044.2 |
| 19:11492406C>A | N/A | c.547G>T  p.(D183Y) | *EPOR* | 24.2 | 0.071 Tolerated | 0.945  Probably Damaging | NM_000121.3  NP_000112.1 |
| 14:24566102C>T | rs768381631 | c.31C>T  (p.L10F) | *PCK2* | 23.4 | 0.0  Damaging | 0.770  Possibly Damaging | NM_004563.3  NP_004554.3 |
| 12:53166642C>G | N/A | c.897G>C  (p.M299I) | *KRT76* | 22.6 | 0.016 Damaging | 0.775  Possibly Damaging | NM_015848.4  NP_056932.2 |
| 7:37934067G>C | rs760527291 | c.1400-1G>C  p.(?) | *NME8* | 21.2 | N/A | N/A | NM_016616.4  NP_057700.3 |
| 16:2201890C>T | N/A | c.150C>T  p.(A449V) | *RAB26* | 18.25 | 0.17  Tolerated | 0.014  Benign | NM_014353.4  NP_055168.2 |

**Table S2** Details of the nine genomic variants identified by WES after filtering and segregation

See Results section for details of how variants were filtered.

Note that the variant in called in *PLEKHM1* (highlighted in grey) could not be confirmed nor checked for segregation with disease due the presence of a near identical pseudogene.

Note that this table does not include CNVs. See Table S3,

^a^ dbSNP, <http://www.ncbi.nlm.nih.gov/projects/SNP/> ([1](#_ENREF_1));

^b^ Combined Annotation Dependent Depletion (CADD) v1.3, <http://cadd.gs.washington.edu/info> ([2](#_ENREF_2));

^c^ SIFT, http://sift.jcvi.org/ ([3](#_ENREF_3));

^d^ PolyPhen2, http://genetics.bwh.harvard.edu/pph2/ ([4](#_ENREF_4));

| **Genomic coordinates (GRCh37)** | **Call** | **Read ratio (number of reads/expected number of reads) and Bayes Factor (BF)** | | | **Gene and exons affected** |
| --- | --- | --- | --- | --- | --- |
|  |  | **II:3** | **III:3** | **IV:2** |  |
| chr19:50463395-50464192 | Heterozygous duplication | 1.66 (308/185)  BF 10.6 | 1.62 (400/247)  BF 13.6 | 1.35 (461/341)  BF 6.56 | *SIGLEC11* exons 2 and 3 (NM_052884.3) |

**Table S3** Details of the one shared copy number variant identified by Exome Depth.

CNVs were analysed from WES data. CNVs that were also shared by three unrelated individuals were discarded.

| **Gene** | **cDNA variant and predicted amino acid change** | **Gene function** | **Animal models** | **Human diseases associated with mutations (inheritance pattern)** | **Gene pLI score (Measure of tolerance for loss of function)** | **Gene missense Z score (measure of tolerance of variation)** |
| --- | --- | --- | --- | --- | --- | --- |
| *SP6* | c.817_818GC>AA  p.(A273K) | Transcription factor | *Sp6*^-/-^ mice reported with little or no enamel, delayed tooth eruption, supernumerary teeth, fused teeth, defected cusp formation, malformed roots and enlarged dentine tubules as well as retarded growth, failure to develop fur and abnormalities in limb development and lung alveolarisation. *Ami*/*Ami* rats with an *Sp6* frameshift mutation (NM_001108833.1: c.965_966insGT, NP_001102303: p.F323Sfs*12) have amelogenesis imperfecta. | None | 0.15 | 1.34 |
| *PLEKHM1* | c.355C>T  p.(R119W) | Lysosomal trafficking and bone homeostasis | Rat “incisors absent” model with *Pleckhm1* frameshift mutation (NM_001009677.1: c.1101delC, NP_001009677: p.L338Sfs*6) has osteopetrosis phenotype. There is generalised skeletal sclerosis, incisors are absent and delayed eruption of other teeth due to defect in bone resorption. | #611497 Osteopetrosis, AR, 6: splice and frameshift mutations identified | 0.01 | 1.18 |
| *CALHM3* | c.671A>C  p.(Q224P) | Calcium channel component | None reported. | Polymorphisms associated with Alzheimer disease, Creutzfeld-Jakob disease. | 0 | 0.35 |
| *EFCC1* | c.419T>G  p.(L140R) | Unknown, bind calcium | None reported. | None reported. | 0 | 0.72 |
| *EPOR* | c.547G>T  p.(D183Y) | Erythropoeitin receptor | No dental phenotype reported. Mice reported with point mutation to constitutively activate the receptor: erythrocytosis and splenomegaly.  Heterozygous mice with truncated mutant human *EPOR* gene had erythrocytosis. *Epor*^-/-^ mice have severe anaemia and die at E13.5. | #133100 erthyrocytosis, familial, 1: AD All reported mutations reside in exon 8 (the variant reported here is in exon 4) and most result in a truncated protein. Note that the variant reported in this study is a missense. | 0.01 | 1.14 |
| *PCK2* | c.31C>T  (p.L10F) | Key enzyme in gluconeogenesis, especially in mitochondrial and hepatic compartments. | None reported. | #261650 PCK2 deficiency, mitochondrial: AR: hypoglycaemia and liver impairment. | 0 | -0.2 |
| *KRT76* | c.897G>C  (p.M299I) | Intermediate filament protein: major structural fibre of epithelial cells. | No dental phenotype reported. *Krt76*^-/-^ mice have flaky tails and dark pigmentation to the tail and footpads. | None reported. | 0 | -0.75 |
| *NME8* | c.1400-1G>C  p.(?) | Outer dynein arm protein of ciliary axoneme. | None reported. | #610852 ciliary dyskinesia, primary, 6: AR with situs ambiguous  Polymorphisms also associated with osteoarthritis, Alzheimer disease and periodontitis | 0 | -0.38 |
| *RAB26* | c.150C>T  p.(A449V) | Intercellular vesicle trafficking, primarily expressed in the brain, target gene of MIST1 that establishes secretory morphology in target cells. | None reported. | None reported. | 0 | -0.81 |
| *SIGLEC11* | Heterozygous duplication: chr19:50463395-50464192 | Cell surface lectin. Mediates anti-inflammatory and immunosuppressive signalling. | None reported. | None reported. | 0 | -0.95 |

**Table S4** Gene function, animal models, associated human diseases and tolerance of variation of the 10 candidate genes identified by WES after filtering and segregation.

Details of human and animal phenotypes were obtained from PubMed and OMIM. Constraint metrics pLI and missense Z scores were obtained from the gnomAD browser v.2.1.1 (accessed 14/11/2019). Note that the variant called in *PLEKHM1* (highlighted in grey) could not be confirmed nor checked for segregation with disease due the presence of a near identical pseudogene.

| **Exon** | **Forward primer (5′-3′)** | **Reverse primer (5′-3′)** | **Size (bp)** | **Genomic coordinates (b37) of exons covered by PCR** | **Genomic coordinates (b37) covered by PCR** |
| --- | --- | --- | --- | --- | --- |
| 1a | CCCCCTTCCCAATTCAGAGA | TTTGTCCCTCTCCCACTGC | 435 | 17:45933020-45933240 | 17:45932905-45933339 |
| 1b | GCGTTCGGAGTGCGTTTTT | AAGGGTTAAAGGCGGCCG | 398 | 17:45928285-45928516 | 17:45928213-45928610 |
| 2_part1 | CAAAGCTGGTGGTCTCCGA | TTTCTGCTTCTTCGCCTCCT | 566 | 17:45924665-45925795 | 17:45925311-45925867 |
| 2_part2 | CGTGGTGGGACCTTCATC | CACAGGGGAACTTCTTGGTG | 594 |  | 17:45924847-45925440 |
| 2_part3 | CAAGACGTCGCACCTGAAG | AATACGCACCTTCCCCTCTT | 440 |  | 17:45924559-45924998 |
| 2_3’UTR_1 | ACATGAGGCTTTCCAAGGGG | CGTGGGTGTAAGATGCCTTT | 500 | 17:45922280-45924598 | 17:45922147-45922646 |
| 2_3’UTR_2 | AGGGGAGCAGAAGAAAACCT | AGGTCTGGGAGCTATATGGG | 486 |  | 17:45922443-45922928 |
| 2_3’UTR_3 | TCTGGTTGGGTTAGCAGGAG | CAAGAGAGGGGCCTTACAGT | 485 |  | 17:45922744-45923228 |
| 2_3’UTR_4 | TGCACACAGGTACACACAAC | GCCCTTGTCTTTCCCTTGC | 499 |  | 17:45923057-45923555 |
| 2_3’UTR_5 | AAGGCCCCTCTCTTGAGTG | TCAGTAGTCTCACATTCCCCA | 600 |  | 17:45923214-45923813 |
| 2_3’UTR_6 | CCTCTGATGAGACACCCCC | GAGATGGGATGGGGGAGC | 498 |  | 17:45923655-45924152 |
| 2_3’UTR_7 | CCCTCCATCTATCTCGGTCC | CCTGCCTCACAGTTTCCCT | 492 |  | 17:45923937-45924428 |
| 2_3’UTR_8 | GGCGCTTTTATGTCCTGGTT | AAAGCCTACGCCAAGACGT | 779 |  | 17:45924231-45925009 |

**Table S5** Primer sequences for Sanger sequencing of *SP6* coding and non-coding exons in individuals with autosomal dominant AI.

**GLI (PDB 2GLI)**

ZF1 ETDCRWDGCSQEFDSQEQLV**H**HINSEHIHGER

ZF2 KEFVCHWGGCSRELRPFKAQYMLV**V**HMRRHTGE

ZF3 KPHKCTFEGCRKSYSRLENLK**T**HLRSHTGE

ZF4 KPYMCEHEGCSKAFSNASDRA**K**HQNRTHSNE

ZF5 KPYVCKLPGCTKRYTDPSSLR**K**HVKTVHG

**ZIF268 (PDB 1ZAA)**

ZF1 HERPYACPVESCDRRFSRSDELT**R**HIRIHTG

ZF2 QKPFQCRICMRNFSRSDHLT**T**HIRTHTG

ZF3 EKPFACDICGRKFARSDERK**R**HTKIHLR

**Figure S1** Alignments of the ZnF sequences for two other Cys2His2 proteins with DNA bound crystal structures deposited in PDB.

Underlined residues are those that bind zinc ions. The residue prior to the first zinc ion-binding His residue is show in bold and is at the equivalent position of SP6 p.Ala273 within each of the zinc fingers.

| **Protein** | **JASPAR reference(s)** | **Position weighted matrices for consensus sequence(s)** | | |
| --- | --- | --- | --- | --- |
| SP1 | MA0079.1  MA0079.2  MA0079.3 | 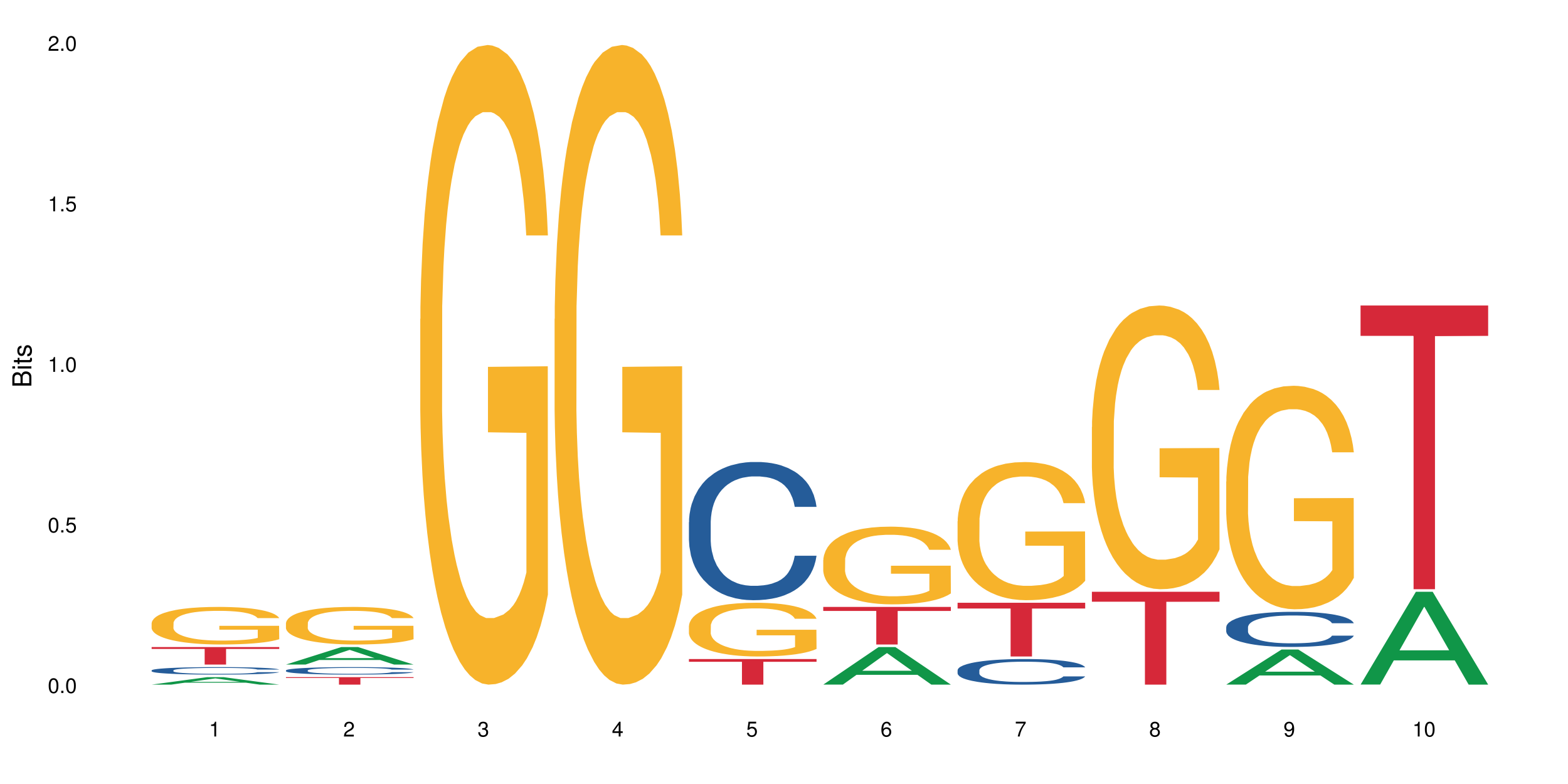 | 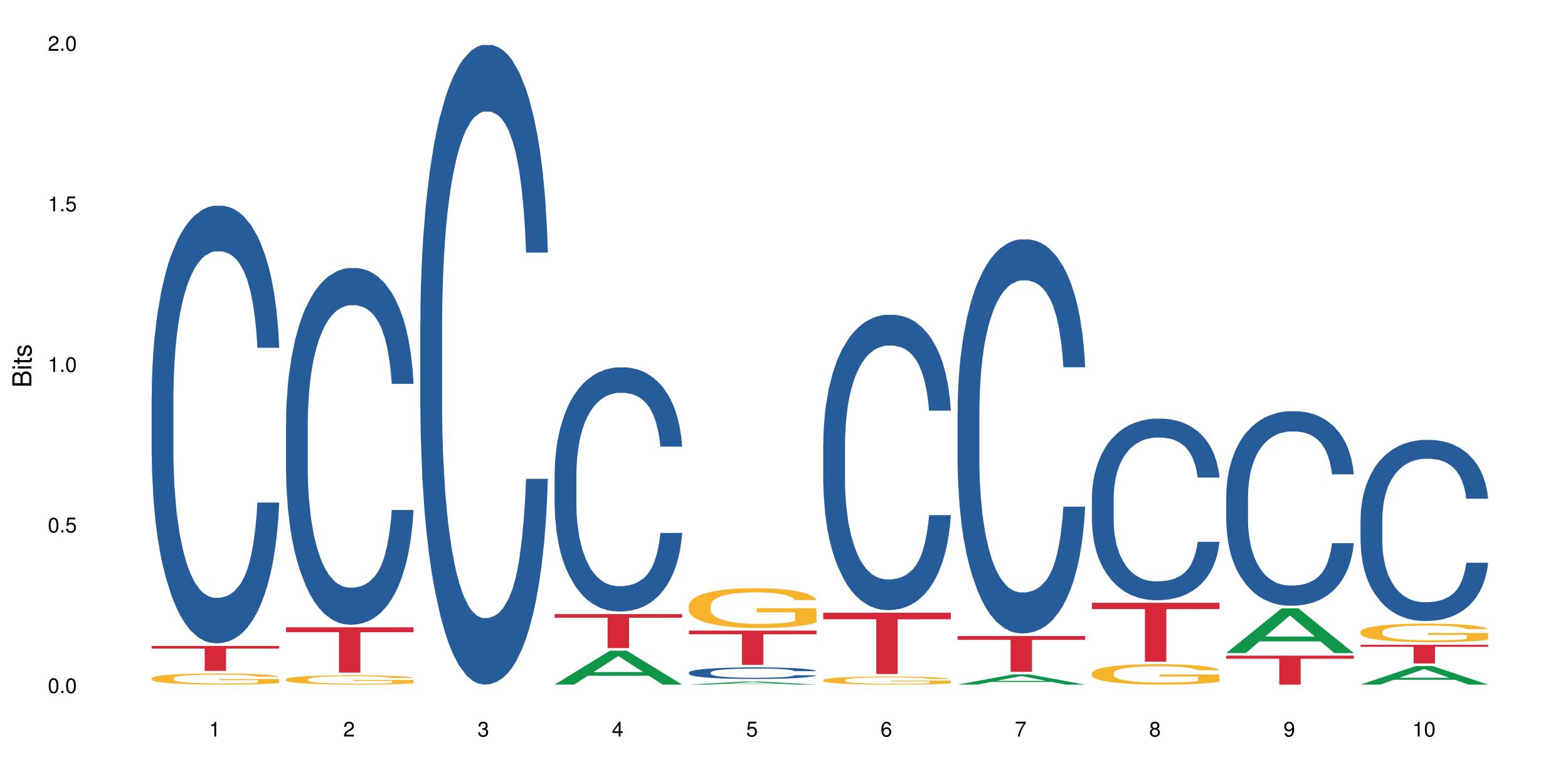 | 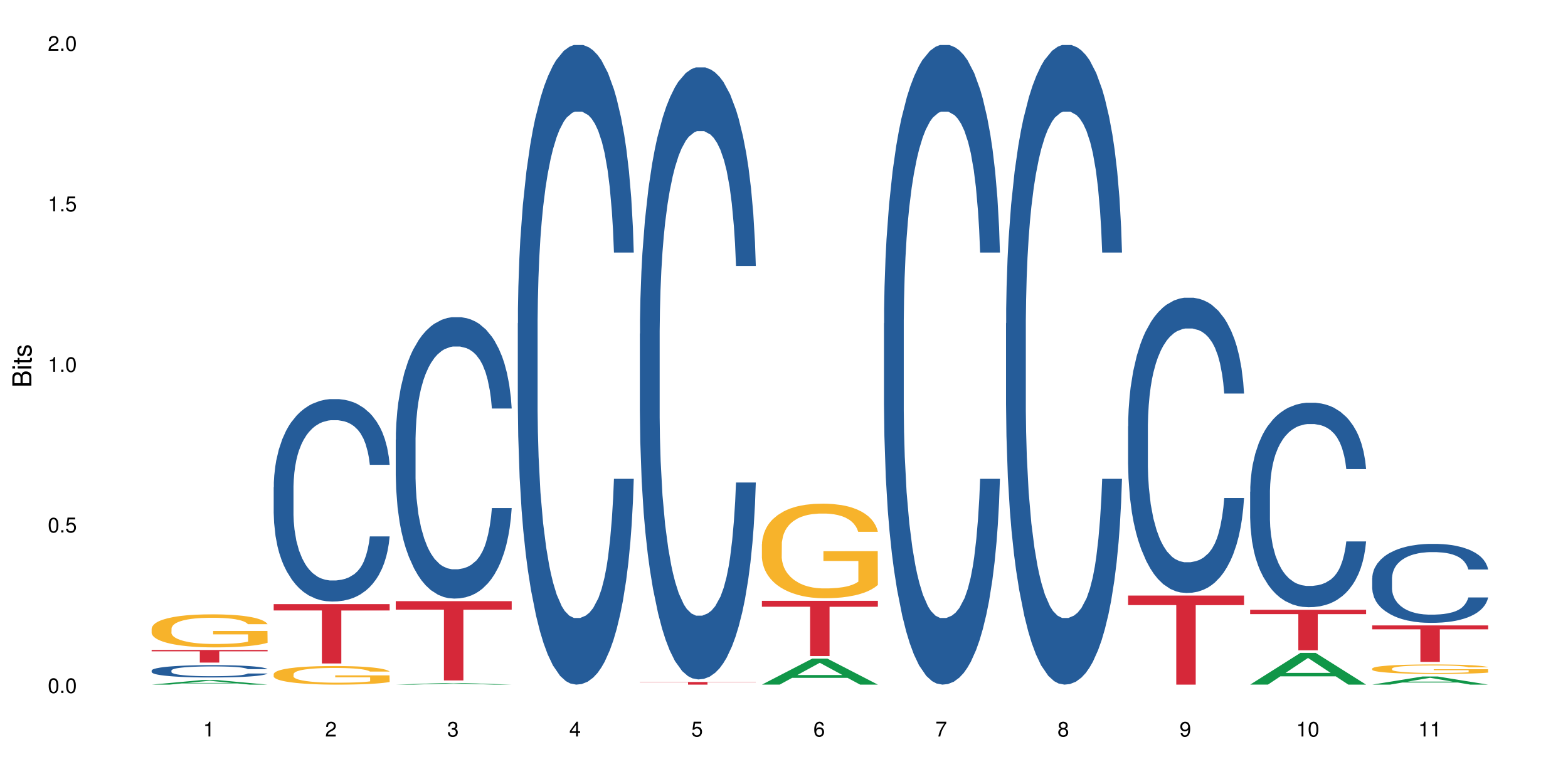 |
| SP2 | MA0516.1 | 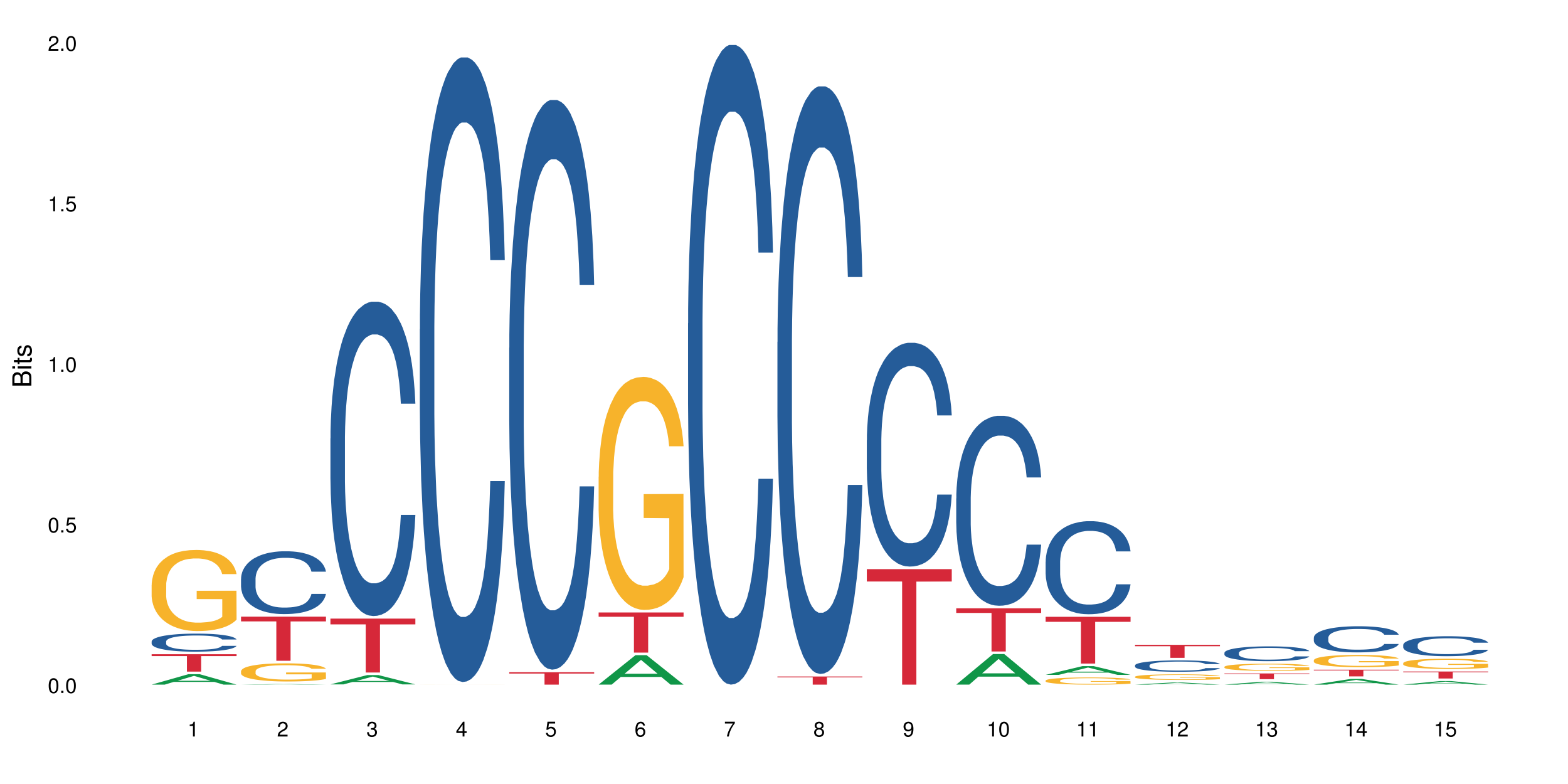 |  |  |
| SP3 | MA0746.1 | 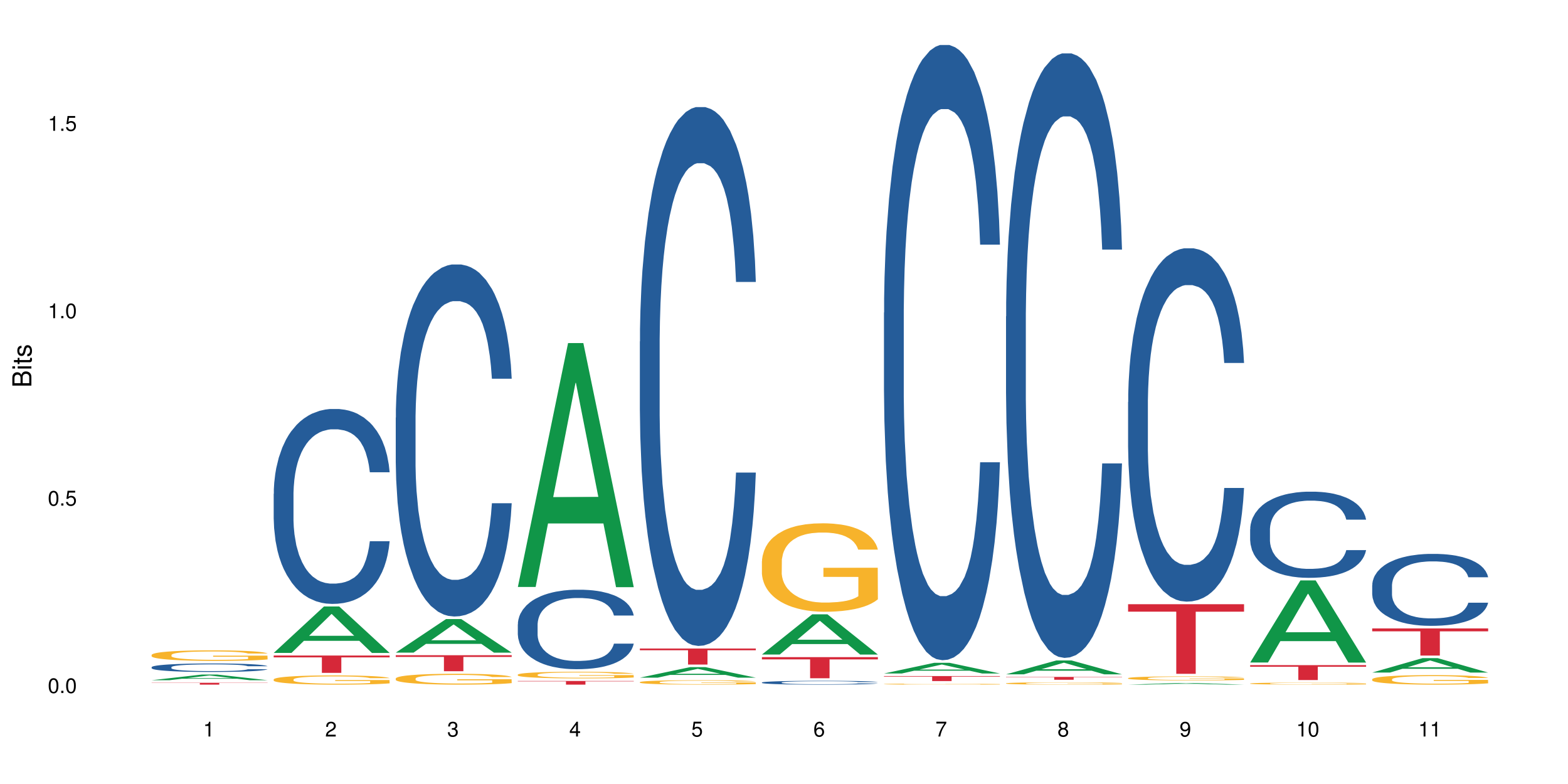 |  |  |
| SP4 | MA0685.1 | 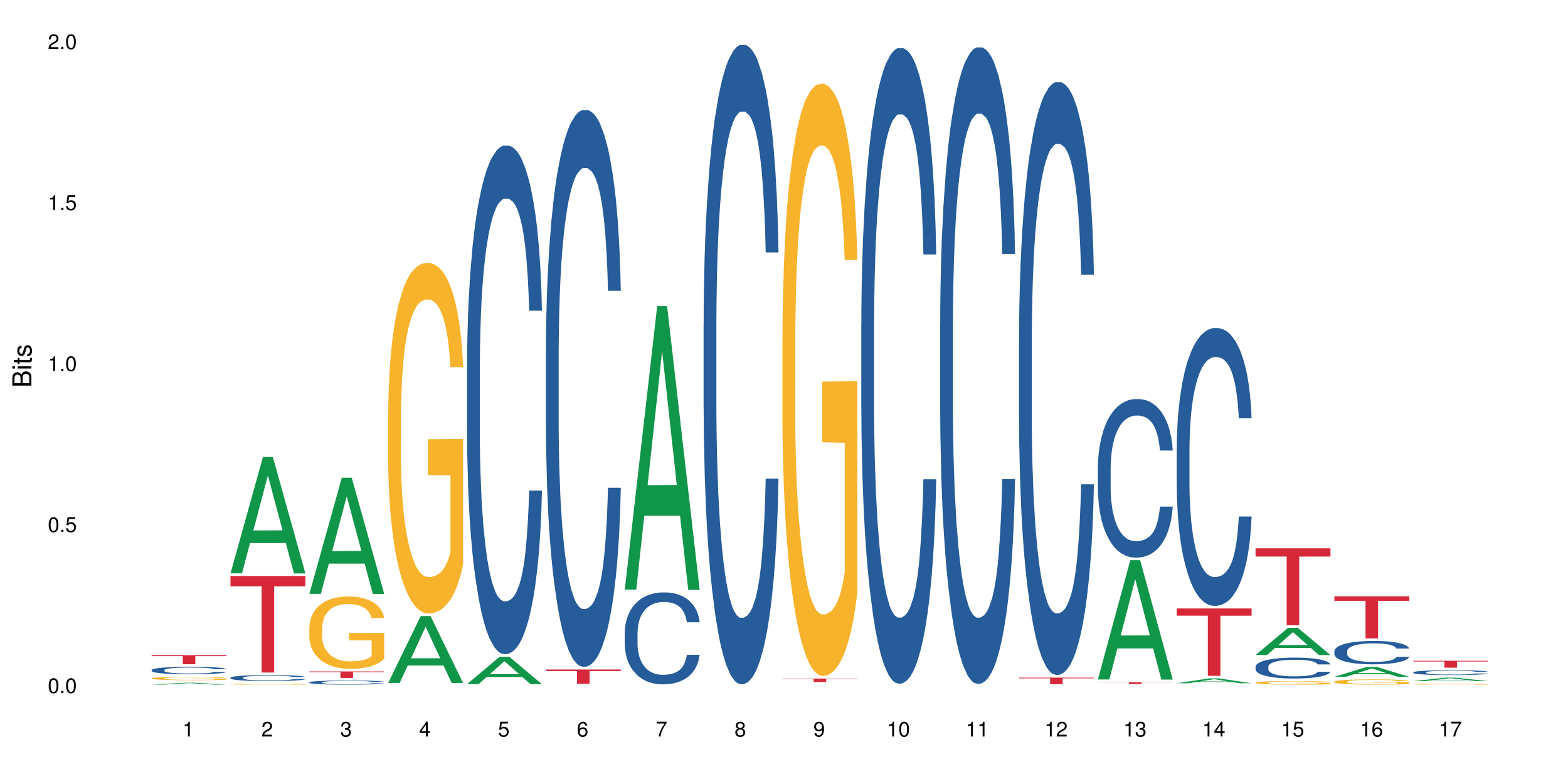 |  |  |
| SP8 | MA0747.1 | 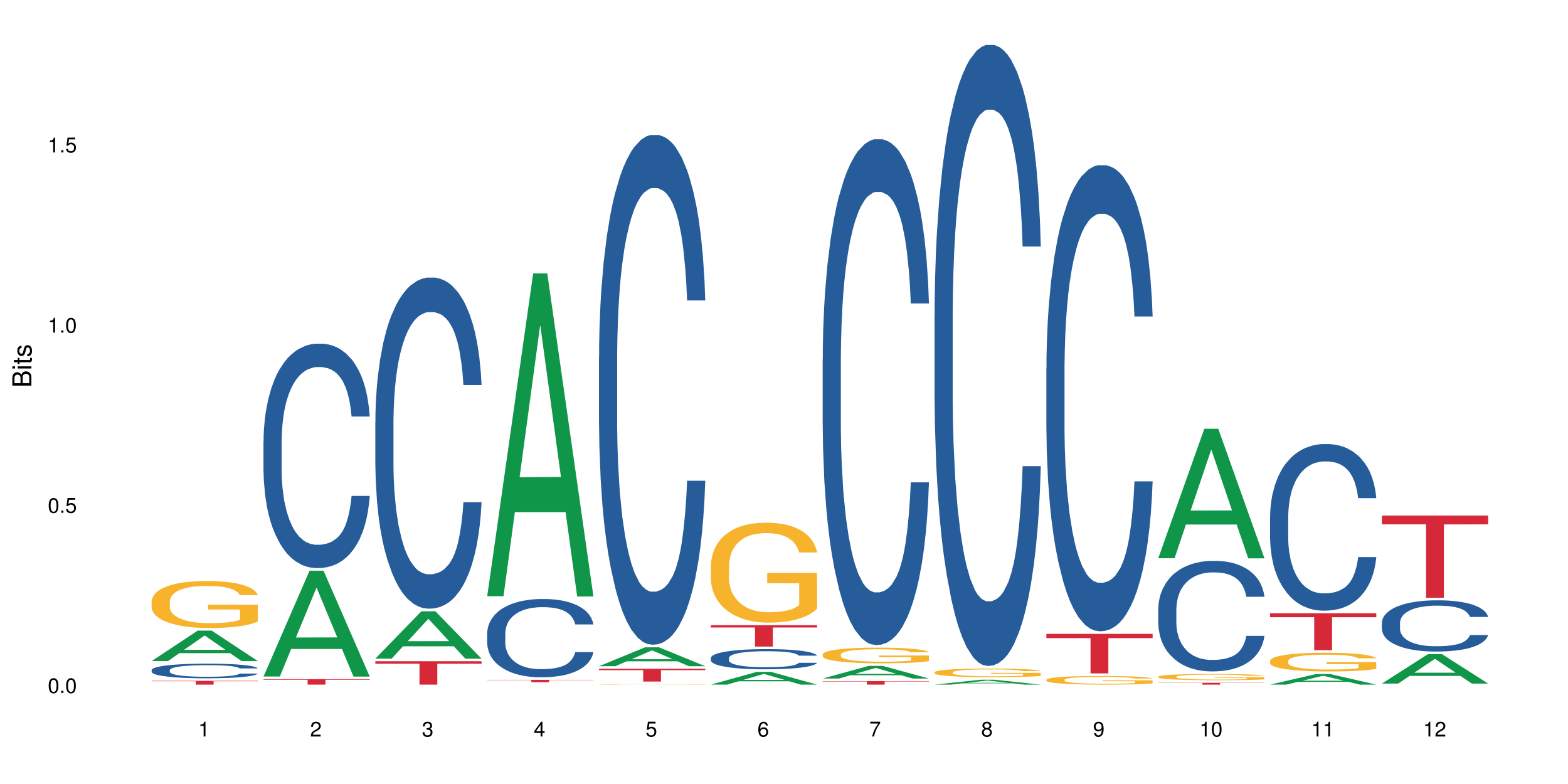 |  |  |

**Table S6** Human SP transcription factor DNA binding site sequences from the JASPAR database.

| **Gene** | **Strand** | **Transcript** | **Coordinates** | **Sequence** |
| --- | --- | --- | --- | --- |
| *ROCK1* | - | NM_005406.3 | chr18:  18691810-18691962 | GAACCTTGCCGGCAGCTGCTTACCCGCACTCGGCTCCTCCCAGCGGTCCGCCTTCTCCACCGCCTGCCCGCAAGGCCCCGCCCACCTCACCGCCCCGCCCCACCCCACCCTGCGGAGGCGACAGAGTGCGCGCGCGCGCGCGGGGTCCCG**GCT** |
| *CA3* | + | NM_005181.4 | chr8:  86350906-86351058 | CCACCTCCGCCCCCGTCACCTCGACAGCTGTCCCGCTCTTGGAATTCATTGGCTTCCTCTACCCGGCCTCCCAAACACCACCCCAATCTAGTTTAGCCCCCCGCCCCACCCTCGCTGACCTAATAAGGCCATGCAGTGTGCGGGGGAGCT**ACA** |
| *FST* | + | NM_013409.2 | chr5:  52776114-52776266 | GAAGTGGGTGTTCTTCCCCACTCCCCACCCCCGACGCGTAGCCCCCAACCCCCGCCCCGGTCGCCTTCCCCCTCCCTCTCGCTCCTACGCAAATAAGAACTCGACGATTCCCTTCCAGCCGTTTTGATTTCGGGCACCTCCGAAAGATAA**TTG** |
| *OSR2* | + | NM_053001.3  and NM_001286841.1 | chr8:  99956481-99956633 | GGAAACCTCGAGCAAACCTGTGCCTTTAAGACTCAGTTGCTATCCAAACAGAAGTAAACAGAGTGGACCATTAGCAGACGCCGGGCGCGGAGGCGGAGCCAGGGCGCTGAGGGCCCCGCGCGGCGGCGGGACGCCCGCCCGAAGGGGAGG**CGG** |
| *PCM1* | + | NM_006197.4 | chr8:  17780347-17780499 | ACTCCGGCGGGTCACATGACTCCAGTCTAGCTCGCATTGCGGCTCCCGCCCGGGCGAGTTCTCGCCCCCGCGCGGCCGTTGCCGAGGAGACGGCGCATGTCCCGCCGCGCGTTGCCCCCTCTGCAGTACCCCCGCCCCTCTTCTCCCACC**ACA** |
| *AMBN* | + | NM_016519.6 | chr4:  71457825-71457977 | CAATGTCCCTGCACGCAATAAAAGGGTGTGGACTAATTGCAGGAGCAGAGATTCCCGCCCCAACTCCCCCCCGCCACCCCTCGCCATTTTGCTTCACTTTGATTGGTGGATTGGCACTTTAAATAAAGGTTTCTAATCTTCCCTGAATGA**GAA** |
| *FAM83H* | - | NM_198488.5 | chr8:  144815947-144816099 | GGGCTGGCGGCGGGCGCGGGGCGCAGGAATGCGGGCGCGCCGCGGGTGGGGCTGCCCCGCCCCCAGGTGCTGCCCGGGCAGGCAGGAGGCAGGAGCGACGGGCGGGGCGGGGGCGGGCGCGTGAATCAGGCCGGGCGGGCCGCGGCAGGG**AGT** |
| *SP6* | - | NM_001258248.2 | chr17:  45928527-45928679 | CTTTCCTCCCCCACTCCCGCCCACCCCCTCCTCGCGGCCCTGGCTGGGCTCCGGTCCAGCCGAGCCCTCAAGGGTTAAAGGCGGCCGCAGGTGAGGTGGGCGGGGCCGCGAGTCCGGGGGAAAAAGCAGCGCTGGGGAGAGGATGAAGGC**AGA** |
| *NKX2-1* | - | NM_001079668.2 | chr14:  36989428-36989580 | GTTGTGGCTCGGGGATCCGGGACA*GCCTCCGGGAGGC*AGTCGATCCCCTACTCAGCGCCCCCTCCGCCCGCTCGGATTCTCTCCGGTAGGGGGAAAGGGGGCGGGGAGCAGAGGTGTCCCTCTGACGGCGGCAGAAGAGAGGCAGACAGA**CTG** |

**Table S7** Comparison of the proximal promoter sequences containing potential SP6 binding sites.

150 bp of sequence prior to each TSS is shown. The first three bases of the TSSs are shown in bold. Putative binding domains for SP6 (CG-rich sequences) are shown in dark grey (9-mer CCCCGCCCC) or light grey (any length). Sequences used for probes are shown underlined. Known SP3 binding sequences are shown in italics.

**Figure S2.** Comparison of DNA-binding activity of wild-type and mutant SP6 proteins using Biacore SPR.

Biotinylated oligonucleotides were captured on a streptavidin–derivatised sensor chip surface. Wild-type and mutant SP6 proteins were washed over these surfaces across a range of concentrations. Each three-minute injection was followed by buffer washes to follow dissociation rates of the SP6-DNA complex. Spikes at 0 and 3 minutes are due to small buffer mismatch effects. Sensorgram results are shown for the *CA3*, *AMBN* and *ROCK1* oligonucleotides (top, middle and bottom respectively). Sequences are presented in Table S8. Protein concentrations are 6.25, 12.5, 25, 50, 100, 200 and 400 nM.

| **Gene** | **Strand** | **Transcript** | **Probe sequence 5′ → 3′** | **Distance from transcriptional start site (+1)** |
| --- | --- | --- | --- | --- |
| *ROCK1* | - | NM_005406.3 | CCCGCCCACCTCACCGCCCCGCCCCACCCCACCCTGCGGA | -74 to -49 |
| *CA3* | + | NM_005181.4 | CAATCTAGTTTAGCCCCCCGCCCCACCCTCGCTGACCTAA | -67 to -28 |
| *AMBN* | + | NM_016519.6 | TGCAGGAGCAGAGATTCCCGCCCCAACTCCCCCCCGCCAC | -113 to -73 |
| *NKX2-1* | - | NM_00317.3 | GGGATCCGGGACA***GCCTCCGGGAGGC***AGTCGATCCCCTAC | -139 to -99 |

**Table S8** Probe sequences used for Biacore surface plasmon resonance studies.

Copies of the 9 bp CCCCGCCCC motif are highlighted yellow with shorter and/or similar sequences in pink. A sequence from the *NKX2-1* promoter, known to bind SP3, was used as a control probe. The sequence bound by SP3 is shown in italics and bold. All probes were biotinylated at the 5′-end.
